# Association between serum Na–Cl level and renal function decline in chronic kidney disease: results from the chronic kidney disease Japan cohort (CKD-JAC) study

**DOI:** 10.1007/s10157-018-1631-x

**Published:** 2018-08-24

**Authors:** Yuichi Maruta, Takeshi Hasegawa, Etsuko Yamakoshi, Hiroki Nishiwaki, Fumihiko Koiwa, Enyu Imai, Akira Hishida

**Affiliations:** 1 Division of Nephrology (Fujigaoka Hospital), Department of Medicine, Showa University School of Medicine, Yokohama, Japan; 2 Office for Promoting Medical Research, Showa University, 1-5-8 Hatanodai, Shinagawa-ku, Tokyo, 142-8555 Japan; 3 Center for Innovative Research for Communities and Clinical Excellence, Fukushima Medical University, Fukushima, Japan; 4 Department of Healthcare Epidemiology, School of Public Health in the Graduate School of Medicine, Kyoto University, Kyoto, Japan; 5 Statcom Company Limited, Tokyo, Japan; 6 Nakayamadera Imai Clinic, Takarazuka, Hyogo Japan; 7 Yaizu City Hospital, Shizuoka, Japan

**Keywords:** Metabolic acidosis, CKD, Electrolyte, Acid–base disorder, Bicarbonate

## Abstract

**Background:**

Metabolic acidosis, which reduces serum bicarbonate levels, contributes to the progression of chronic kidney disease (CKD). The difference between sodium and chloride (Na–Cl) may theoretically predict serum bicarbonate levels. This study aimed to evaluate serum Na–Cl level as a risk factor for renal function decline among patients who participated in the chronic kidney disease Japan cohort (CKD-JAC) study.

**Methods:**

The association between low Na–Cl concentration (< 34 mmol/L) and composite renal function decline events (any initiation of renal replacement therapy or 50% decline in estimated glomerular filtration rate) was evaluated among 2143 patients with CKD stage G3a-4. Using Cox regression analysis, hazard ratios (HRs) were estimated after adjusting for the following covariates: age, sex, diabetes mellitus, diabetic nephropathy, cardiovascular disease, anemia, angiotensin-converting enzyme inhibitors and angiotensin II receptor antagonists, loop diuretics, cigarette smoking, body mass index, serum albumin, systolic blood pressure, urine albumin-to-creatinine ratio, and CKD stage.

**Results:**

Composite renal function decline events were observed in 405 patients (18.9%) over the 4-year follow-up period. Low serum Na–Cl level (< 34 mmol/L) was independently associated with a greater risk for composite renal function decline events (HR 1.384; 95% confidence interval [CI], 1.116–1.717). Subgroup analyses identified that the association between low Na–Cl level and composite renal function decline events was stronger among patients with CKD stage G4 and those with anemia.

**Conclusions:**

Our investigation suggests that Na–Cl is an independent predictor of CKD progression, especially among patients with CKD stage G4 and those with anemia.

**Electronic supplementary material:**

The online version of this article (10.1007/s10157-018-1631-x) contains supplementary material, which is available to authorized users.

## Introduction

Metabolic acidosis is a well-known complication of chronic kidney disease (CKD), particularly in stages 4 and 5, and reduces the serum level of sodium bicarbonate, which is important for attenuating the rate of kidney disease progression [1–7]. According to the Kidney Disease Improving Global Outcomes (KDIGO) 2012 Clinical Practice Guideline for the Evaluation and Management of Chronic Kidney Disease, the serum bicarbonate level in patients with CKD should be 22 mmol/L or higher [8]. Alternately, the Japanese Society of Nephrology (JSN) suggests using the difference between sodium and chloride levels (Na–Cl) as a surrogate marker of metabolic acidosis [9].

In theory, the Na–Cl level corresponds to the serum bicarbonate level, except in the event of anion accumulation and hypoalbuminemia. Especially, in Japan, the serum bicarbonate concentration is calculated from pH and partial arterial carbon dioxide pressure (pCO_2_) using the Henderson–Hasselbalch equation. A system for directly measuring the venous serum bicarbonate concentration was established only in 2015. However, its use is still not prevalent because of the lack of coverage by Japanese insurance. Therefore, another metabolic acidosis marker, such as the Na–Cl level, has been utilized. However, the suitability of the Na–Cl level as a prognostic marker for CKD aggravation has never been investigated in prospective clinical trials intended for CKD patients.

This study aimed to confirm whether a low serum Na–Cl level is associated with a higher risk for a decline in renal function among patients in the pre-dialysis phase of CKD.

## Materials and methods

### Design and participants

This was a retrospective cohort study using the data of CKD-JAC study. The protocol of the CKD-JAC study has been published previously, with relevant features summarized as follows [10]. The CKD-JAC study was a multicenter cohort study that enrolled 2966 Japanese patients followed up by nephrologists from 17 selected medical institutions in Japan. The inclusion criteria were Japanese patients living in Japan who were 20–75 years old and with an estimated glomerular filtration rate (eGFR) of 10–59 mL/min/1.73 m^2^ at baseline. The exclusion criteria were patients with polycystic kidney disease, human immunodeficiency virus infection, cirrhosis, active cancer, or cancer treatment in the past 2 years; transplant recipients; patients with a history of previous or current dialysis; pregnant patients; and patients who did not provide informed consent. In addition to these previously published exclusion criteria [10], four additional exclusion criteria were used in our ad hoc analysis: (1) an eGFR < 15 mL/min/1.73 m^2^ at baseline, as this has been reported to reflect anion accumulation [11], (2) missing sodium or chloride concentration at baseline, (3) malignancy, and (4) a serum albumin level < 3.0 g/dL, as low Na–Cl levels may be mistakenly observed in patients with hypoalbuminemia [12, 13].

### Exposures

The KDIGO practice guidelines recommend maintaining the serum bicarbonate at a minimum level of 22 mmol/L, which generally means maintaining the Na–Cl concentration at a minimum of 34 mmol/L [8]. The utility of Na–Cl as a surrogate marker of serum levels of bicarbonate is based on the following formula: anion gap (AG) = Na^+^ − (Cl^−^ + HCO_3_^−^); thus, HCO_3_^−^ + AG = Na^+^ − Cl^−^. Normally, if no extra anions such as lactate or ketone bodies are present, the AG is approximately 12 [4]. For the determination of Na–Cl levels, blood was drawn from a vein, and collected and examined at a central laboratory. Subsequently, patients were categorized into groups based on their Na–Cl levels at baseline: low (< 34 mmol/L) and normal (≥ 34 mmol/L) groups.

### Endpoint

The primary endpoint was defined as a composite of the initiation of any renal replacement therapy (RRT) or a significant decline in the eGFR from baseline. A significant decline in the eGFR was defined as a decrease in the serum creatinine (Cre) value ≥ 50% from baseline on three consecutive measurements, with the day of the last measurement considered to be the endpoint. The eGFR was calculated using the following equation: for male, eGFR (mL/min/1.73 m^2^) = 194 × [age] − 0.287 × [serum Cre (mg/dL)]− 1.094, and for female, eGFR (mL/min/1.73 m^2^) = 194 × [age]− 0.287 × [serum Cr (mg/dL)]− 1.094 × 0.739 [14].

### Covariates

The following were initially used as clinical factors related to Na–Cl and outcomes: diabetes mellitus (DM) was defined as an increase ≥ 6.5% in HbA1c (glycated hemoglobin) or an antidiabetic medication use. Diabetic nephropathy was defined by the nephrologist in charge. Cardiovascular disease (CVD) was defined as the occurrence of myocardial infarction, angina pectoris, or cerebrovascular event. Cigarette smoking was defined as the current use of cigarettes. Serum albumin, serum hemoglobin (Hb), urine albumin, and urine creatinine were examined at a central laboratory. Body mass index (BMI) was calculated as follows: weight (kg)/[height (m) × height (m)]. CKD stages were divided into three stages: G3a, G3b, and G4, based on the CKD guidelines [8].

### Statistical analyses

Continuous variables and categorical variables were expressed as median ± interquartile range (IQR) and proportion, respectively. Comparison between normal and low Na–Cl groups was evaluated using Student’s *t* test or chi-squared test, as appropriate for the distribution of the data. The Cox regression model was used to detect the baseline characteristics associated with the primary endpoint. All covariates, age, sex, DM, diabetic nephropathy, CVD, angiotensin-converting enzyme inhibitors/angiotensin II receptor antagonists (ACEIs/ARBs), loop diuretics, cigarette smoking, serum albumin, BMI, systolic blood pressure (SBP), urine albumin-to-creatinine ratio (UACR), Hb, and CKD stage, were included in the model. The missing values of covariates were multiply-imputed in the primary analysis assuming that the missing values were at random (MAR). Multiple imputation was used to handle missing data for the primary outcome analyses [15]. The results across 100 imputed data sets were combined by averaging, and standard errors (SEs) were adjusted to reflect both within-imputation variability and between-imputation variability. The estimates and their SEs were combined using Rubin’s rules. In addition, a Cox regression model that considered death as the competing risk was used to estimate the hazard ratio (HR) [16]. Secondary analyses were conducted with the addition of other potentially important variables that were not included as covariates in the primary analysis because of their low frequency [use of thiazide, potassium-sparing diuretics, and erythropoiesis-stimulating agents (ESA)]. To confirm the stability of Na–Cl correlation to metabolic acidosis and the reliability under the hypoalbuminemia, sensitivity analyses were also conducted for the following two conditions: a change in the Na–Cl cut-off value from 34 to 32 mmol/L and for serum albumin levels dichotomized as < 4.0 g/dL or ≥ 4.0 g/dL, used instead of the measured value, to correct for the AG alternation, and therefore, the calculated Na–Cl level [Na–Cl + 2.5 × (4-Alb)]. In addition, two other subgroup analyses were performed using Cox regression analysis. The first subgroup analysis was used to determine the effect of CKD stage when Na–Cl is directly associated with the endpoints, while the other was used to evaluate the specific effect of anemia, defined by Hb level < 12 g/dL. Covariates were included in the Cox regression models for these subgroups analyses, including age, sex, diabetic nephropathy, ACEIs/ARBs, serum albumin, SBP, and UACR. A two-tailed *P* value < 0.05 was considered statistically significant. All statistical analyses were performed using the SAS software (version 9.4; SAS Institute Inc., Cary, NC, USA).

## Results

### Baseline characteristics

**Fig. 1:**
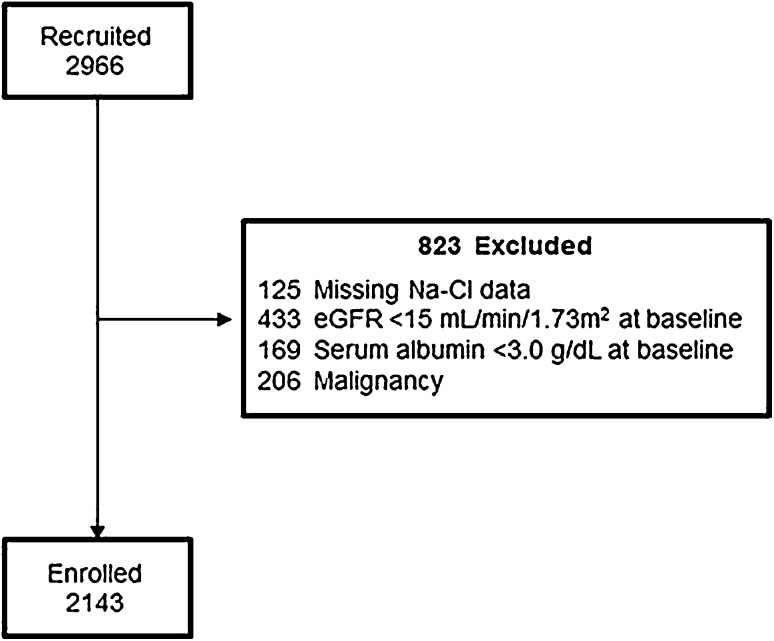
Participant enrollment

The patient flow diagram is shown in Fig. 1. After excluding 121 patients, because of the absence of baseline data, withdrawal of consent, or loss to follow-up, 2966 patients enrolled in the CKD-JAC study in September 2007 were eligible. Those patients without Na and Cl data (125), patients with a eGFR < 15 ml/min/1.73 m^2^ at baseline (433), patients with serum albumin level < 3.0 g/dL (169), and patients with history of malignancy (206) were excluded. After screening, 2143 patients were enrolled in our study, with a study end time of March 2013.

The characteristics of the CKD-JAC study participants according to a Na–Cl concentration of < 34 mmol/L (low group, comprising 39.5% of the study population) or ≥ 34 mmol/L (normal group) are presented in Table 1. At baseline, group differences were observed in the serum albumin level, UACR, Hb level, eGFR, use of ACEIs/ARBs, use of loop diuretics, a history of CVD, and DM. Data regarding the use of erythropoiesis-stimulating agents (ESA), thiazide, and potassium-sparing diuretics are shown in Supplemental Table 1; these factors were prescribed too infrequently in the study population to be evaluated in the primary analysis.

**Table 1:** Patients’ characteristics at baseline

Characteristics	Total		Na–Cl			
			≥ 34 mmol/l		< 34 mmol/l	
	*n* = 2143	Missing *n* (%)	*n* = 1296	Missing *n* (%)	*n* = 847	Missing *n* (%)
Male (%)	61.5	0 (0)	60	0 (0)	63.8	0 (0)
Age (yr)	62 (53–69)	0 (0)	63 (54–70)	0 (0)	61 (52–69)	0 (0)
DM nephropathy (%)	18.7	25 (1.2)	18.8	14 (1.1)	18.5	11 (1.3)
DM (%)	35.8	0 (0)	37.9	0 (0)	32.7	0 (0)
History of CVD (%)	22.3	0 (0)	24.2	0 (0)	19.4	0 (0)
ACEIs/ARBs (%)	20.3	0 (0)	17.4	0 (0)	24.6	0 (0)
Loop diuretics (%)	20.6	0 (0)	22.6	0 (0)	17.6	0 (0)
Cigarette smoking (%)	14.3	320 (14.9)	13	201 (15.5)	16.4	119 (14.4)
BMI (Male %)	23.7 (21.6–26.0)	124 (5.8)	23.6 (21.6–26.1)	83 (6.4)	23.8 (21.6–25.9)	41 (4.8)
BMI (Female %)	22.5 (20.1–25.4)	132 (4.5)	22.7 (20.3–25.4)	71 (5.5)	22.1 (19.7–25.4)	32 (3.8)
Alb (g/dl)	4.0 (3.8–4.3)	0 (0)	4.1 (3.9–4.3)	0 (0)	3.9 (3.7–4.2)	0 (0)
Systolic blood pressure (mmHg)	130 (119–142)	32 (1.5)	130 (120–142)	27 (2.1)	130 (118–141)	5 (0.6)
UACR (mg/g・Cre)	423 (99–1135)	204 (9.5)	322 (68–960)	129 (10.0)	576 (179–1369)	75 (8.9)
Hb (g/dl)	12.2 (11.1–13.4)	26 (1.2)	12.5 (11.4–13.9)	22 (1.7)	11.7 (10.7–12.8)	4 (0.5)
eGFR(ml/min/1.73 m^2^)	30.9 (22.4–39.5)	0 (0)	34.3 (25.8–41.6)	0 (0)	25.5 (19.5–34.5)	0 (0)

All data shown with median (25–75% quartiles)

*DM* diabetes mellitus, *CVD* cardiovascular disease, *ACEIs* angiotensin-converting enzyme inhibitors, *ARBs* angiotensin receptor blockers, *BMI* body mass index, *Alb* serum albumin, *UACR* urine albumin-to-creatinine, *Hb* hemoglobin

### Composite renal function decline events in the CKD-JAC cohort

**Fig. 2:**
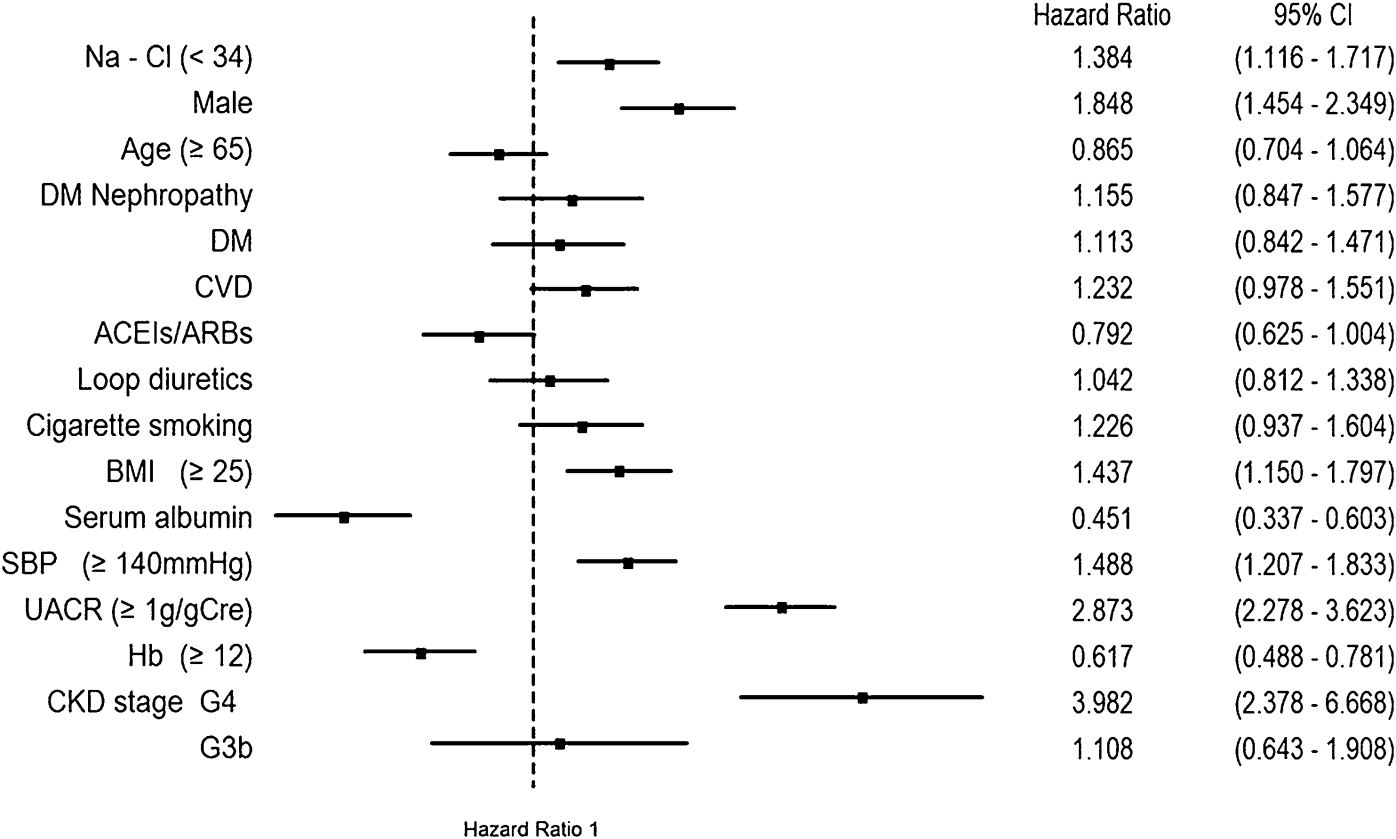
Associations of evaluated variables with composite renal function decline events in Japanese patients with CKD stage G3a-4. Composite renal function decline event: any initiation of renal replacement therapy or a 50% decline in the eGFR from baseline; *DM* diabetes mellitus, *CVD* cardiovascular disease, *ACEIs* angiotensin-converting enzyme inhibitors, *ARBs* angiotensin receptor blockers, *BMI* body mass index, *SBP* systolic blood pressure, *UACR* urine albumin-to-creatinine ratio, *Hb* hemoglobin

Among the 2143 patients with CKD stage G3-4, a composite renal function decline event (any initiation of RRT or 50% decline in the eGFR from baseline over the 4-year observation period) was identified in 405 patients (18.9%). The association between the Na–Cl level and the composite outcome was evaluated using a Cox regression model (Fig. 2). The low Na–Cl concentration was associated with the composite outcome after covariate adjustments (age, sex, DM, diabetic nephropathy, CVD, ACEIs/ARBs, loop diuretics, cigarette smoking, BMI, serum albumin, SBP, UACR, Hb, and CKD stages; HR 1.384; 95% confidence interval [CI], 1.116–1.717). The HR was comparable when using death as the competing risk (HR 1.428; 95% CI 1.108–1.840). Furthermore, this result was robust to the addition of thiazide, potassium-sparing diuretics, and ESA treatment to the covariates (HR 1.396; 95% CI 1.125–1.733; Supplemental Fig. 1). The two sensitivity analyses confirmed the stability of this primary endpoint using a Na–Cl cut-off value of 32 mmol/L (HR 1.351; 95% CI 1.036–1.763) and when using a corrected Na–Cl level for patients with a serum albumin < 4.0 g/dL (HR 1.306; 95% CI 1.025–1.662).

### Composite renal function decline events among patients with different CKD stages

Among the CKD stages (G3a, G3b, and G4), a low Na–Cl concentration was associated with composite renal function decline events (any initiation of RRT or 50% decline in the eGFR from baseline) only among patients with CKD stage G4 in the Cox regression model adjusted for the covariates (HR 1.444; 95% CI 1.093–1.906; Table 2).

**Table 2:** Associations of variables with composite renal function decline events in Japanese patients: differences based on CKD stages

Variables	Stage G3a (165)		Stage G3b (651)		Stage G4 (699)	
	HR (95% CI)	*P* value	HR (95% CI)	*P* value	HR (95% CI)	*P* value
Na–Cl (< 34 mmol/L)	3.630 (0.827–15.934)	0.088	1.194 (0.664–2.149)	0.554	1.444 (1.093–1.906)	0.010
Male	3.178 (0.344–29.352)	0.308	2.824 (1.444–520)	0.002	1.966 (1.449–2.666)	< 0.001
Age (≥ 65 years)	1.034 (0.176–6.066)	0.971	0.878 (0.495–1.556)	0.655	0.941 (0.721–1.228)	0.655
Diabetic nephropathy	1.950 (0.398–9.542)	0.410	1.622 (0.894–2.945)	0.112	1.301 (0.960–1.764)	0.090
ACEIs/ARBs	1.330 (0.316–5.600)	0.698	1.197 (0.639–2.242)	0.575	0.680 (0.495–0.935)	0.018
Serum albumin (g/dL)	1.628 (0.240–11.049)	0.618	0.286 (0.136–0.602)	0.001	0.427 (0.295–0.618)	< 0.001
Systolic blood pressure (≥ 140 mmHg)	1.051 (0.252–4.376)	0.946	1.518 (0.860–2.680)	0.150	1.574 (1.198–2.067)	0.001
UACR (≥ 1000 mg/g・Cre)	14.435 (2.014–103.471)	0.008	2.981 (1.572–5.651)	< 0.001	3.23 (2.407–4.335)	< 0.001

Composite renal function decline event: any initiation of RRT or 50% decline in eGFR from baseline. All data shown with hazard ratio (95% confidence interval)

*DM* diabetes mellitus, *ACEIs* angiotensin-converting enzyme inhibitors, *ARBs* angiotensin receptor blockers, *UACR* urine albumin-to-creatinine ratio

### Composite renal function decline events based on hemoglobin concentration

A low Na–Cl concentration was associated with composite renal function decline events (any initiation of RRT or 50% decline in the eGFR from baseline) in the group with anemia (Hb < 12 g/dL) (HR 1.517; 95% CI 1.089–2.115; Table 3). This association was not found in the group without anemia.

**Table 3:** Associations of variables with composite renal function decline events in Japanese patients: differences based on hemoglobin concentration

	Hb			
Variables	≥ 12 g/dL		< 12 g/dL	
	HR (95% CI)	*P* value	HR (95% CI)	*P* value
Na–Cl (< 34 mmol/L)	1.182 (0.801–1.744)	0.400	1.517 (1.089–2.115)	0.014
Male	1.892 (1.120–3.197)	0.017	2.528 (1.808–3.537)	< 0.001
Age (≥ 65 years)	0.776 (0.517–1.165)	0.221	0.918 (0.677–1.245)	0.583
Diabetic nephropathy	1.364 (0.886–2.100)	0.159	1.287 (0.921–1.800)	0.140
ACEIs/ARBs	0.992 (0.650–1.515)	0.971	0.711 (0.493–1.027)	0.069
Serum albumin (g/dL)	0.312 (0.181–0.537)	< 0.001	0.568 (0.375–0.861)	0.008
Systolic blood pressure (≥ 140 mmHg)	1.232 (0.836–1.817)	0.292	1.798 (1.322–2.446)	< 0.001
UACR (≥ 1000 mg/g・Cre)	4.494 (2.909–6.943)	< 0.001	2.889 (2.069–4.034)	< 0.001
CKD stage G4	6.066 (2.432–15.129)	< 0.001	3.537 (1.427–8.764)	0.006
G3b	1.795 (0.696–4.631)	0.226	0.980 (0.370–2.598)	0.968

Composite renal function decline event: any initiation of RRT or 50% decline in eGFR from baseline. All data shown with hazard ratio (95% confidence interval)

*DM* diabetes mellitus, *ACEIs* angiotensin-converting enzyme inhibitors, *ARBs* angiotensin receptor blockers, *UACR* urine albumin-to-creatinine ratio

## Discussion

The present study shows that a low Na–Cl level is independently associated with CKD progression, particularly among patients with an eGFR between 15 and 30 mL/min/1.73 m^2^ and among those with anemia (Hb < 12 g/dL). Metabolic acidosis has been shown to be associated with worsening renal disease in humans [1–3]. Furthermore, previous studies have reported that bicarbonate supplementation slows the progression of kidney disease, if optimum serum bicarbonate level is maintained [2, 5, 6, 17]. One of the previous studies reported that relative to the reference group (bicarbonate level, 25–26 mmol/L), the hazard ratio for 50% reduction of the eGFR was 1.54 (95% CI 1.13–2.09) for bicarbonate levels of 22 mmol/L or less [1]. This bicarbonate level (22 mmol/L) was applicable to 34 mmol/L in Na–Cl. Na–Cl was not equivalent to the serum bicarbonate level, but Na–Cl was reported as a simple parameter for acid–base status assessment, and the correlation coefficient was 0.733 in that study [18]. This supports our results; therefore, any of our outcomes may be associated with an acid–base disorder.

Hypoalbuminemia is an important factor that causes a dissociation between the Na–Cl level and the serum bicarbonate level, as the AG decreases to 2.5 mmol/L when the serum albumin level decreases to 1 mmol/L [12, 13]. Thus, in this study, severe hypoalbuminemia (< 3.0 g/dL) was excluded, and an additional analysis was attempted which attenuated the effect of hypoalbuminemia. Specifically, when the measured albumin value was < 4.0 g/dL, a corrected Na–Cl level, calculated as Na–Cl + 2.5 × (4-observed serum albumin), was used to categorize cases into low and normal Na–Cl groups. This correction confirmed the association between Na–Cl and renal function decline: (HR 1.428; 95% CI 1.114–1.831 for the measured Na–Cl level and HR 1.306; 95% CI 1.025–1.662 for the corrected Na–Cl level). This association, however, was only assured among patients with a serum albumin ≥ 3.0 g/dL. In the CKD-JAC cohort, only 2.5% of patients presented with severe hypoalbuminemia (< 3.0 g/dL). Thus, the use of the measured Na–Cl value was applicable in most of the CKD patients in our cohort.

When other anions are produced in the body, this correlation between the Na–Cl level and serum bicarbonate level is no longer reliable, but typically, an anion quickly disappears physiologically. Thus, in this cohort, temporary anion production would be less important than chronic metabolic acidosis. The use of the Na–Cl level as a marker of metabolic acidosis is only accurate within the normal range of the AG, as the sum of the serum bicarbonate level and the AG is equivalent to the Na–Cl level. In other words, a normal range of the AG is necessary for a normal range of the Na–Cl level. Thus, we included patients with CKD stages 3–4, as these stages have reported AGs in the normal range, and excluded patients with CKD stage 5, as this stage has a reported AG of 16.02 ± 0.66 [11]. Additionally, the newer auto-analyzers measure a higher serum chloride level, resulting in a lower AG, as reported by Winter et al. [19]. Consequently, the normal range of Na–Cl might actually be lower than the range we estimated. Considering this point, we completed a sensitivity analysis using a lower Na–Cl cut-off value of 32 mmol/L to categorize patients into low and normal Na–Cl groups. The similar trend as for the cut-off value of 34 mmol/L was identified in this analysis (HR 1.351; 95% CI 1.036–1.763). Clarifying the exact normal value of the Na–Cl level is beyond the scope of this study, as only a few patients had a Na–Cl level < 30 mmol/L in our study cohort. Therefore, maintaining a Na–Cl concentration ≥ 32 mmol/L would be reasonable for our cohort.

In general, metabolic acidosis is indicative of the inability of the kidneys to synthesize ammonia, regenerate bicarbonate, and excrete hydrogen ions, resulting in a decrease in Na–Cl levels. This pathway might explain the association between lower Na–Cl levels and poor renal outcomes and increased risk of tubulointerstitial injury [20]. Using an animal model, Nath et al. proposed that tubulointerstitial damage could be caused by the activation of the complement cascade due to an increase in renal cortical ammonia [21]. This hypothesis might explain why patients who maintain a normal serum bicarbonate level, either naturally and/or by supplementation, have a better Na–Cl level and, correspondingly, better kidney survival.

In CKD stage G4 (eGFR 15–30 mL/min/1.73 m^2^), a low Na–Cl level was strongly associated with poor outcomes (Table 2). de Brito-Ashurst et al. reported on the effectiveness of bicarbonate supplementation in slowing the progression of CKD among patients with a creatinine clearance of 15–30 mL/min/1.73 m^2^ and serum bicarbonate concentration of 16–20 mmol/L [5]. Over a 2-year follow-up, supplementation with 600-mg sodium bicarbonate tablets among these patients was sufficient to maintain a serum bicarbonate level ≥ 23 mmol/L. Moreover, the rate of end-stage renal disease (ESRD) development was lower among those who received bicarbonate supplementation (6.5%) than among those who did not receive supplementation (33%). Therefore, bicarbonate supplementation reduced the relative risk of ESRD (0.13; 95% CI 0.04–0.40). In our cohort, patients with CKD stage G4 who maintained a Na–Cl concentration ≥ 34 mmol/L had a better kidney prognosis, regardless of sodium bicarbonate supplementation status.

Interestingly, Hb concentration influenced the association between Na–Cl level and the incidence rate of renal function decline (Table 3), with only anemic patients having a poor outcome in kidney function. Generally, Hb plays an important buffering role against acidosis, incorporating free H^+^ ions into CO_2_ to form HCO_3_^−^ [22]. Therefore, anemia impairs this buffering system, resulting in poor renal survival among patients with a low Na–Cl level. Improving anemia in patients with CKD may attenuate this aspect of CKD progression.

This study is the first to report on the direct association between the Na–Cl level and renal function decline. The Na–Cl level was indicated as the surrogate marker for the serum bicarbonate level, but was not shown as the renal function decline marker. This result suggested that both physiological and physicochemical approaches are possible for CKD patients.

This study has some limitations. First, the effect of diuretics on the measured association between the Na–Cl level and renal function decline was not sufficiently analyzed. Spironolactone blocks the actions of aldosterone, and amiloride reduces Na^+^ ion reabsorption in the collecting duct, which results in hyporeninemic hypoaldosteronism [23]. Although these diuretics may have had an influence on the incidence of composite renal function decline events, the association between a low Na–Cl level and composite renal function decline events remained significant in the secondary analysis, which controlled for the use of these diuretics (Supplemental Fig. 1). Second, the association between pre-supplementation serum bicarbonate and Na–Cl levels in this study was not known, because the CKD-JAC study checked serum bicarbonate level only in the patients who were prescribed sodium bicarbonate (9% of all patients). Third, this study enrolled only Japanese outpatients cared for by nephrologists belonging to the medical institutions that treat CKD patients in each area. Thus, there may be selection bias.

In conclusion, the Na–Cl level is a simple parameter for an acid–base disorder without arterial blood gas examination, and our investigation shows that the Na–Cl level is an independent predictor of CKD progression, especially among patients with CKD stage G4 and those with anemia.

## Electronic supplementary material

Below is the link to the electronic supplementary material.

Supplementary material 1 (DOCX 17 KB)

Supplementary material 2 (PDF 67 KB)
