# Supplementary material for: Association between serum Na–Cl level and renal function decline in chronic kidney disease: results from the chronic kidney disease Japan cohort (CKD-JAC) study
Source: Clin Exp Nephrol. 2018 Aug 24;23(2):215–22. doi: 10.1007/s10157-018-1631-x (PMC6510908; doi:10.1007/s10157-018-1631-x)
Supplement: Supplementary file 1 — Supplementary material 1 (DOCX 17 KB) [file 10157_2018_1631_MOESM1_ESM.docx]

Sup. Table 1. Patients characteristics at baseline

|  | Total | |  | Na-Cl | | | | |
| --- | --- | --- | --- | --- | --- | --- | --- | --- |
| Characteristics |  | |  | ≥ 34mmol/l | |  | <34mmol/l | |
|  | n=2143 | missing n(%) |  | n=1296 | missing n(%) |  | n=847 | missing n(%) |
| Male (%) | 61.5 | 0(0) |  | 60 | 0(0) |  | 63.8 | 0(0) |
| Age (yr) | 62 (53-69) | 0(0) |  | 63 (54-70) | 0(0) |  | 61 (52-69) | 0(0) |
| DM nephropathy (%) | 18.7 | 25(1.2) |  | 18.8 | 14(1.1) |  | 18.5 | 11(1.3) |
| DM (%) | 35.8 | 0(0) |  | 37.9 | 0(0) |  | 32.7 | 0(0) |
| History of CVD (%) | 22.3 | 0(0) |  | 24.2 | 0(0) |  | 19.4 | 0(0) |
| ACEIs/ARBs (%) | 20.3 | 0(0) |  | 17.4 | 0(0) |  | 24.6 | 0(0) |
| Loop diuretics (%) | 20.6 | 0(0) |  | 22.6 | 0(0) |  | 17.6 | 0(0) |
| Thiazide (%) | 7.3 | 0(0) |  | 6.8 | 0(0) |  | 8.1 | 0(0) |
| Potassium-sparing diuretics (%) | 6.1 | 0(0) |  | 6.0 | 0(0) |  | 6.3 | 0(0) |
| Cigarette smoking (%) | 14.3 | 320(14.9) |  | 13 | 201(15.5) |  | 16.4 | 119(14.4) |
| BMI (Male %） | 23.7 (21.6-26.0) | 124(5.8) |  | 23.6 (21.6-26.1) | 83(6.4) |  | 23.8(21.6-25.9) | 41(4.8) |
| BMI (Female %) | 22.5 (20.1-25.4) | 132(4.5) |  | 22.7 (20.3-25.4) | 71(5.5) |  | 22.1 (19.7-25.4) | 32(3.8) |
| Alb (g/dl) | 4.0 (3.8-4.3) | 0(0) |  | 4.1 (3.9-4.3) | 0(0) |  | 3.9 (3.7-4.2) | 0(0) |
| Systolic blood pressure (mmHg) | 130 (119-142) | 32(1.5) |  | 130 (120-142) | 27(2.1) |  | 130 (118-141) | 5(0.6) |
| UACR (mg/g・Cre) | 423 (99-1135) | 204(9.5) |  | 322 (68-960) | 129(10.0) |  | 576 (179-1369) | 75(8.9) |
| Hb (g/dl) | 12.2 (11.1-13.4) | 26(1.2) |  | 12.5 (11.4-13.9) | 22(1.7) |  | 11.7 (10.7-12.8) | 4(0.5) |
| ESA (%) | 8.4 | 0(0) |  | 5.2 | 0(0) |  | 13.3 | 0(0) |
| eGFR(ml/min/1.73m2) | 30.9 (22.4-39.5) | 0(0) |  | 34.3 (25.8-41.6) | 0(0) |  | 25.5 (19.5-34.5) | 0(0) |

DM, Diabetes mellitus; CVD, cardiovascular disease; ACEIs, angiotensin-converting enzyme inhibitors; ARBs, angiotensin receptor blockers; BMI, body mass index;

Alb, serum albumin; UACR, urine albumin-to-creatinine; Hb, hemoglobin; ESA, erythropoiesis-stimulating agent.

All data shown with median (25%–75% quartiles)
