# Supplementary figures and images for: Association between serum Na–Cl level and renal function decline in chronic kidney disease: results from the chronic kidney disease Japan cohort (CKD-JAC) study
Source: Clin Exp Nephrol. 2018 Aug 24;23(2):215–22. doi: 10.1007/s10157-018-1631-x (PMC6510908; doi:10.1007/s10157-018-1631-x)

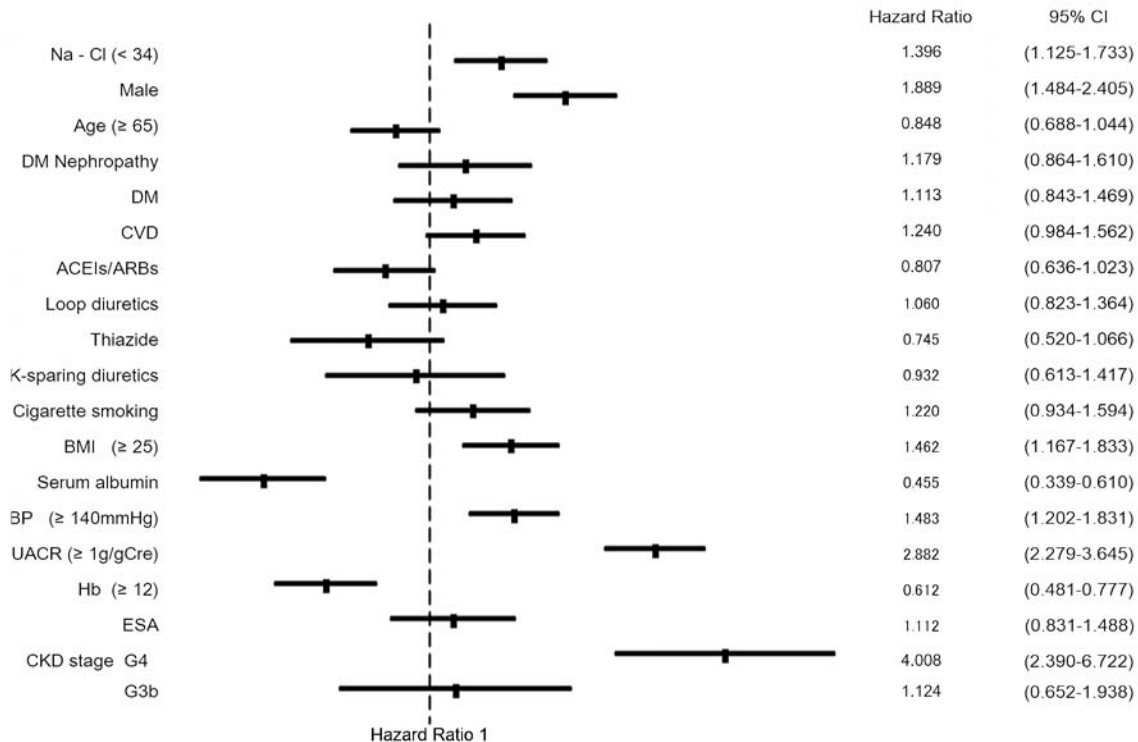

Supplement: Supplementary file 2 — Supplementary material 2 (PDF 67 KB) [file 10157_2018_1631_MOESM2_ESM.pdf]
